# Aberrant methylation of *PCDH10* and *RASSF1A* genes in blood samples for non-invasive diagnosis and prognostic assessment of gastric cancer

**DOI:** 10.7717/peerj.2112

**Published:** 2016-06-09

**Authors:** Charinya Pimson, Tipaya Ekalaksananan, Chamsai Pientong, Supannee Promthet, Nuntiput Putthanachote, Krittika Suwanrungruang, Surapon Wiangnon

**Affiliations:** 1 Biomedical Science Programme, Graduate School, Khon Kaen University,Khon Kaen,Thailand; 2 Department of Microbiology, Faculty of Medicine, Khon Kaen University,Khon Kaen,Thailand; 3 HPV & EBV and Carcinogenesis Research Group, Khon Kaen University,Khon Kaen,Thailand; 4 Department of Epidemiology, Faculty of Public Health, Khon Kaen University,Khon Kaen,Thailand; 5 Cancer Unit, Srinagarind Hospital, Faculty of Medicine, Khon Kaen University,Khon Kaen,Thailand; 6 Department of Pediatrics, Faculty of Medicine, Khon Kaen University,Khon Kaen,Thailand

**Keywords:** Gastric cancer, *PCDH10*, *RASSF1A* methylation, Survival, Clinicopathological factors

## Abstract

**Background.** Assessment of DNA methylation of specific genes is one approach to the diagnosis of cancer worldwide. Early stage detection is necessary to reduce the mortality rate of cancers, including those occurring in the stomach. For this purpose, tumor cells in circulating blood offer promising candidates for non-invasive diagnosis. Transcriptional inactivation of tumor suppressor genes, like *PCDH10* and *RASSF1A*, by methylation is associated with progression of gastric cancer, and such methylation can therefore be utilized as a biomarker.

**Methods.** The present research was conducted to evaluate DNA methylation in these two genes using blood samples of gastric cancer cases. Clinicopathological data were also analyzed and cumulative survival rates generated for comparison.

**Results.** High frequencies of *PCDH10* and *RASSF1A* methylations in the gastric cancer group were noted (94.1% and 83.2%, respectively, as compared to 2.97% and 5.45% in 202 matched controls). Most patients (53.4%) were in severe stage of the disease, with a median survival time of 8.4 months after diagnosis. Likewise, the patients with metastases, or *RASSF1A* and *PCDH10* methylations, had median survival times of 7.3, 7.8, and 8.4 months, respectively. A Kaplan–Meier analysis showed that cumulative survival was significantly lower in those cases positive for methylation of *RASSF1A* than in their negative counterparts. Similarly, whereas almost 100% of patients positive for *PCDH10* methylation had died after five years, none of the negative cases died over this period. Notably, the methylations of *RASSF1A* and *PCDH10* were found to be higher in the late-stage patients and were also significantly correlated with metastasis and histology.

**Conclusions.** *PCDH10* and *RASSF1A* methylations in blood samples can serve as potential non-invasive diagnostic indicators in blood for gastric cancer. In addition to *RASSF1A* methylation, tumor stage proved to be a major prognostic factor in terms of survival rates.

## Introduction

Gastric cancer is in the top three causes of cancer mortality worldwide (Lin, Huang & Juan, 2012) and in 2012 was the fifth most common cancer with more than 70% occurring in developing countries and with especially high incidences in Eastern Asia. Rates in men are generally twice those in women (Ferro et al., 2014). Although advances in treatment can help prolong patient life, mortality rates are still high in many countries because established cancer screening programs are limited and presentation is very often at the late stage (Hamashima et al., 2015). While there exist several novel screening techniques available for early detection, such as testing for pepsinogens and *H. pylori* factors in the circulation (Miki et al., 2003; Miki, 2011; Liu et al., 2014; Pasechnikov et al., 2014), these strategies may not be appropriate in a relatively low risk country like Thailand.

In general, blood samples can be particularly useful in cancer screening by, for example, a proteomics approach (Li et al., 2012a; Li et al., 2012b). Both circulating tumor cells and cell free DNA may have prognostic value (Ignatiadis & Dawson, 2014; Madic et al., 2015). Although challenges exist, effective methodology has been established (Coumans et al., 2012; Saucedo-Zeni et al., 2012), and the search is on for serum cancer markers with good sensitivity and specificity (Hung, Chiu & Lo, 2009; Kohler et al., 2011; Sayres & Cho, 2011). There are several reports that DNA methylation can be applied with tumor tissues harvested after surgical operation or biopsy for prediction of prognosis. Moreover, its presence in serum offers clear advantages for non-invasive detection. DNA methylation plays an important role in silencing tumor suppressor genes during cancer development by adding a methyl group from S-adenosyl-L-methionine to the cytosine or adenine ring in CpG islands of genes (Levenson, 2010; Warton & Samimi, 2015). DNA methylation can thus suppress the transcription of many tumor suppressor genes protecting against cancer initiation and progression (Jones & Baylin, 2007), and thus offer tools for screening.

The *PCDH10* gene is classified as a tumor suppressor gene in the protocadherin family, a cadherin subfamily. *PCDH10* function specifies cell–cell adhesion via Ca^2+^ in tissue morphogenetic processes (Almeida et al., 2010; Otani et al., 2013) and apoptosis by upregulation of *Fas*, *Caspase 8*, *Jun*, *CDKN1A*, and *HTATIP2* (Yu et al., 2009). The methylation of *PCDH10* is involved in metastasis and has been found in many carcinomas, including colorectal, nasopharyngeal, esophageal, hepatocellular, breast, cervical, and lung cancers, and also in gastric cancer (Li et al., 2012a; Li et al., 2012b; Deng et al., 2014). The RAS association domain family 1A gene (*RASSF1A*) or *RASSF1A* blocks cell-cycle progression and inhibits cyclin D1 accumulation. Furthermore, RAS regulates a pro-apoptotic pathway by binding to the RAS effectors, NORE1 and *RASSF1A*, and activates apoptotic protein kinases such as MST1 (Dammann et al., 2003). *RASSF1A* is also classified as a tumor suppressor gene, and its methylation may lead to increased cell proliferation, invasion, and metastasis (Hesson, Cooper & Latif, 2007).

There have been reports about alteration of either *PCDH10* or *RASSF1A* in gastric cancer tissues or cell lines (Byun et al., 2001; Dammann et al., 2003; Shi et al., 2014), but none of the studies investigated both genes together. In addition, to our knowledge, only a few specifically addressed the methylation of *RASSF1A* in DNA extracted from peripheral blood (Balgkouranidou et al., 2015; Wang et al., 2008). Thus, the aim of this research was to assess the methylation status of *PCDH10* and *RASSF1A* of DNA in blood samples of gastric cancer patients. This study also included an investigation of clinicopathological characteristics affecting the survival rate of this patient group in northeastern Thailand.

## Materials and Methods

### Population and specimens

Specimens were obtained from gastric cancer patients who visited two of the largest hospitals in the northeast of Thailand (Srinagarind Hospital at Khon Kaen University—a medical school hospital, and Khon Kaen Hospital—a regional public hospital) and had resided in the area for at least five years. The patients were initially diagnosed by the specialist and confirmed by histopathology and diagnosed according to the International Classification of Diseases for Oncology in the period between October 2002 and 2006. All patients were followed-up until death or the end of the study period (31 October, 2012). This study was approved by the Khon Kaen University Ethics Committee for Human Research (HE 581260, dated 20 July, 2015). General demographic characteristics, such as age and sex, were assessed along with clinicopathological parameters of gastric cancer.

EDTA blood samples were collected by venipuncture from 101 patients diagnosed with gastric cancer according to the International Classification of Diseases for Oncology (ICD-O 3rd) and confirmed by histopathology. Each case was matched with two controls (a total of 202) by gender, age (±3 years), hospital, and provincial residence. The participants with gastrointestinal disease were excluded. All participants provided written informed consent prior to the beginning of the study. The participants who refused, or unable to answer our interview were excluded from the study.

Plasma was separated by centrifugation at 2000 × g for 15 min at room temperature. All samples were stored at −20 °C until DNA extraction. All patients were followed-up until death or the end of the study period (31 October, 2012). Data of interest were retrieved from medical records including age on the day of diagnosis, gender, site of cancer, histopathology, histological grading, stage of disease, and tumor metastasis. The classical endpoint was survival time. The clinicopathological status of each patient was checked from medical records and by linkage with the death registry of the Thai national statistics database.

### DNA extraction

The cell-free DNA was extracted from plasma using the standard protocol of the Genomic DNA Mini Kit (Geneaid Biotech). Briefly, 200 µl of plasma was mixed with 200 µl of working solution and incubated at 60 °C for 10 min. The DNA isolation was then processed as described in the manufacturer’s protocol. DNA samples were stored at −80 °C until used.

### Gene methylation by methylation-specific polymerase chain reaction

Methylation-specific PCR (MSP) was used to evaluate methylation status based on bisulfite reactivity. Firstly, DNA was denatured to create single-stranded DNA and then modified with sodium bisulfite followed by a PCR using two pairs of primers: (1) specific for methylated DNA and (2) specific for unmethylated DNA. Bisulfite modification was used to perform the conversion of unmethylated cytosine to uracil except for the 5-methylcytosines. Bisulfite conversion was carried out up to 2 µg of extracted DNA in accordance with the instructions of EpiTect Bisulfite Kit (Qiagen, Hilden, Germany). PCR reactions were carried out in a total volume of 25 µl per 1 reaction containing 2.5 µl of 10X PCR buffer, 0.5 µl of 50 mM Mgcl_2_, 1 µl of 10 mM dNTP, 0.5 µl of forward primer (10 µM), 0.5 µl of reverse primer (10 µM), and 1 µl of template modified DNA.

To investigate the methylation of *PCDH10* promoters, the orders of bases in unmethylation and methylation of *PCDH10* forward and reverse primers were as follows: 5′-GTTGTT AAATAGATATGTTATGT-3′ and 5′-CTAAAAACTAAAAACTTTCCACA-3′; 5′-TCGTTA AATAGATACGTTACGC-3′, and 5′-TAAAAACTAAAAACT TTCCGCG-3′, respectively. MSP conditions were setup as follows: a hot start at 95 °C for 15 min followed by 55 cycles of 95 °C for 30 s, 59 °C for 30 s, 72 °C for 30 s, and finally 72 °C for 8 min (final extension).

The sequences of specific primers in unmethylation of *RASSF1A* promoters (forward and reverse primers) were as follows: 5′ GGTTTTGTGAGAGTGTGTTTAG-3′ and 5′-CACTAACAAACACAAACCAAAC-3′; and 5′-GGGTTTTGCGAGAGCGCG-3′, and 5′-GCTAACAAAGCGGAACCG-3′ for *RASSF1A* methylation, respectively. The condition of MSP was setup as follows: a hot start at 95 °C for 15 min followed by 55 cycles of 95 °C, for 30 s, 59 °C for 30 s, 72 °C for 30 s, and finally 72 °C for 8 min (final extension). Positive methylated DNA was human peripheral blood leukocyte DNA which was treated with 75 units of M.SssI methylase, and positive unmethylated was non-treated DNA.

The amplified 150 bp products from the methylated and unmethylated *PCDH10* and *RASSF1A*were run on 2% agarose gel with 1,000 bp DNA ladder, and stained with ethidium bromide and visualized in UV light.

### Statistical analysis

Statistical comparisons of prevalence data were performed using the Fisher’s exact test. Where indicated, *p* < 0.001 is denoted ***; 0.001 < *p* < 0.01 is denoted **, and 0.01 < *p* < 0.05 is denoted *. Survival times were calculated for each patient and started from the date of diagnosis until the date of the death or the end of following-up period (31 October, 2012). Percentages were used to describe categorical data. Means with standard deviations or medians with ranges were used to describe continuous data characteristics. The survival probabilities were determined by the Kaplan–Meier method. Comparisons were made with the log-rank test. Univariate and multivariate Cox proportional hazard regression models were used to estimate the association between explanatory variables and survival experience, and the results presented in the form of crude and adjusted hazard ratios (HR) and their 95% confidence intervals (CI). All analyses were conducted using Stata version 10.0 (http://www.stata.com/). *P*-values are two-tailed and reported without any formal correction for multiple comparisons.

**Table 1:** Influence of clinicopathological factor, *RASSF1A* and *PCDH10* methylation on survival of 101 gastric cancer patients.

Variable	Number (%)	Median survival (months)	Crude HR (95%CI)	*p*-value	Adjusted HR 95%CI	*p*-value
Gender						
Female	44(43.6)	7.8(5.7−12.3)	1			
Male	57(56.4)	10.2(6.1−14.3)	0.81(0.53−1.23)	0.33	–	–
Age (years)						
≤40	17(16.8)	8.8(1.9−19.8)	1			
41–50	25(24.7)	8.5(4.6−12.4)	0.61(0.14−2.63)			
51–60	29(28.7)	8.3(6.7−20.8)	0.43(0.10−1.84)			
≥61	30(29.7)	7.5(5.4−18.8)	0.44(0.10−1.90)	0.30	–	–
Region of cancer						
Upper site (cardia and fundus)	18(17.8)	12.8(4.3−20.2)	1			
Body	7(6.9)	11.4(5.1−21.3)	0.88(0.34−2.23)			
Lower site (antrum and pyrolus)	48(47.5)	8.6(5.7−14.3)	1.00(0.57−1.77)	0.65	–	–
Histopathology						
Adenocarcinoma (NOS)	70(69.3)	8.7(5.7−12.8)	1			
Adenocarcinoma (intestinal and diffuse type)	31(30.7)	8.6(6.0−16.5)	0.93(0.59−1.45)	0.76	–	–
Histology grading (Goseki classification)						
Group I, II (well or moderate differentiation)	21(20.8)	8.8(4.8−21.3)	1			
Group III, IV (poor differentiation)	59(58.4)	8.7(6.7−13.0)	1.08(0.64−1.82)	0.65	–	–
Stage grouping (TNM classification)						
Mild and moderate stage (IA, IB, II, IIIA, IIIB)	23(22.8)	17.3(6.8−39.1)	1			
Severe stage (IV)	54(53.4)	8.6(5.7−11.5)	2.56(1.45−4.52)^***^	0.001	2.62(1.74-3.97)^***^	0.001
Metastasis						
No	33(32.7)	11.5(6.7−16.5)	1			
Yes	68(67.3)	7.3(4.5−8.8)	1.76(1.13−2.74)^**^	0.01	1.09(0.69−1.71)	0.77
*RASSF1A* methylation						
Unmethylation	17(16.8)	20.2(5.9−22.6)	1			
Methylation	84(83.2)	7.8(5.8−10.2)	2.96(1.52−5.76)^***^	0.001	2.33(1.14-4.85)^**^	0.002
*PCDH10* methylation						
Unmethylation	6(5.9)	NA	1			
Methylation	95(94.1)	8.4(6.1−11.4)	7.23e+s15(NA)	NS	–	–

**Notes.**

*** *p* < 0.001.

** 0.001 < *p* < 0.01.

Note that for some groupings, it is possible that the information cannot be used as indicated: the region of cancer not known in 28 patients, and this also applies for histology grading (21 patients), and stage grouping (24 patients).

## Results

**Figure 1:**
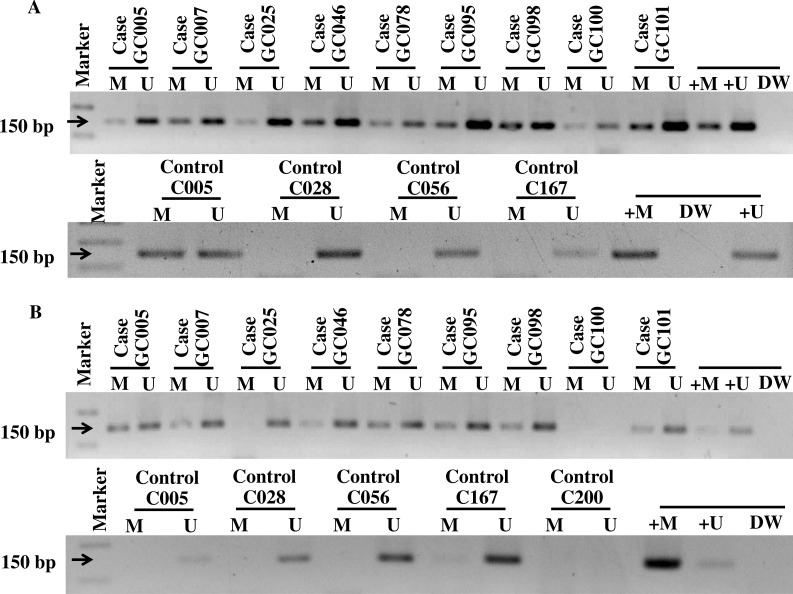
The gel electrophoresis of MS-PCR products for methylated (M), unmethelated (U), positive methylated (+M), normal saline (DW), and positive unmethylated (+U) in gastric cancer patients and controls; *PDCH10* (A) and *RASSF1A* (B).

**Figure 2:**
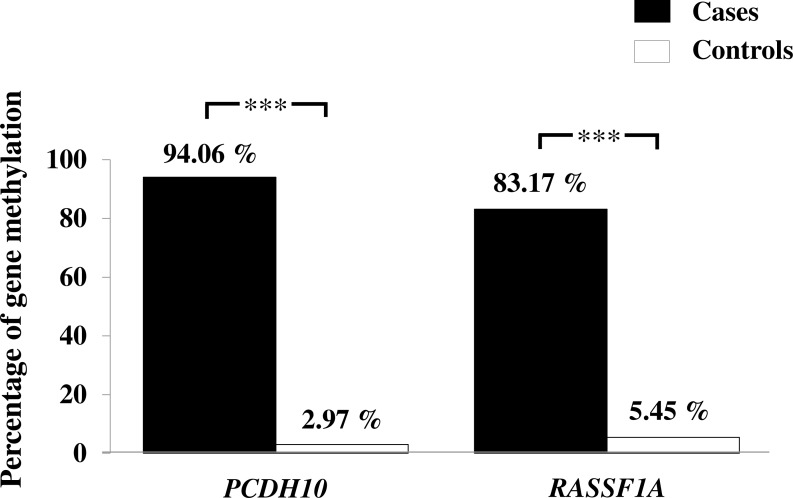
Assessment of methylation status of *PCDH10* and *RASSF1A* genes in gastric cancer patients and controls by MSP where indicated, *p* < 0.001 is denoted ***; 0.001 < *p* < 0.01 is denoted **, and 0.01 < *p* < 0.05 is denoted *.

**Figure 3:**
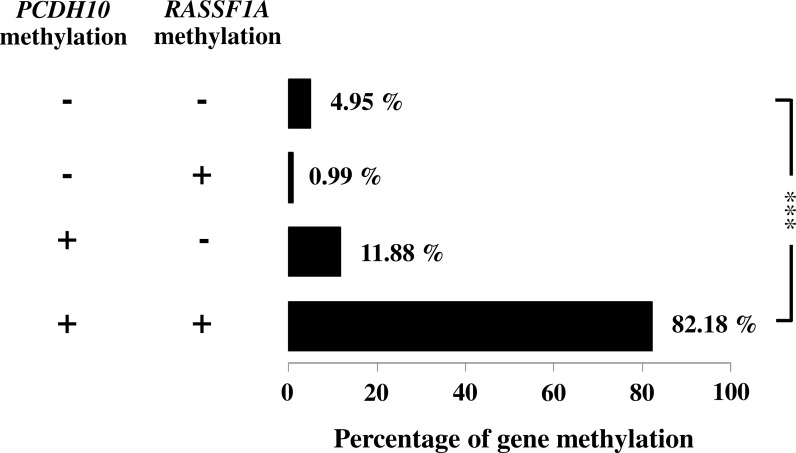
Association of *PCDH10* and *RASSF1A* gene methylations in gastric cancer patients; (−, −) is both unmethylated; (−, +) is the only methylated *RASSF1A*; (+, 1) is the only methylated *PCDH10*; and (+, +) is both methylated where indicated, *p* < 0.001 is denoted ***; 0.001 < *p* < 0.01 is denoted **, and 0.01 < *p* < 0.05 is denoted *.

General characteristics of the gastric cancer patients (101), such as gender and age, and frequencies of clinicopathological variables are summarized in Table 1. Findings for the methylation status are shown in Fig. 1. With both *PCDH10* and *RASSF1A*, methylation was very rare in controls (2.97% and 5.45%, respectively), but exceedingly common in cancer cases (94.06% in *PCDH10* and 83.17% in *RASSF1A*) with highly significant differences (*p* < 0.001) using the chi-square test (Fig. 2). In the cases group, a total of 83 demonstrated methylation of both genes with only one having *RASSF1A* alone as compared to 12 for *PCDH10* alone (Fig. 3).

### Survival rates for gastric cancer cases

**Figure 4:**
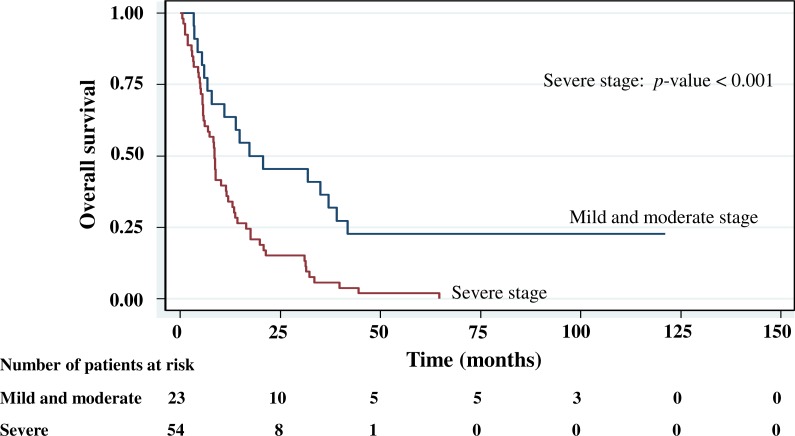
Survival curve of gastric cancer by stage of cancer status (*n* = 101).

**Figure 5:**
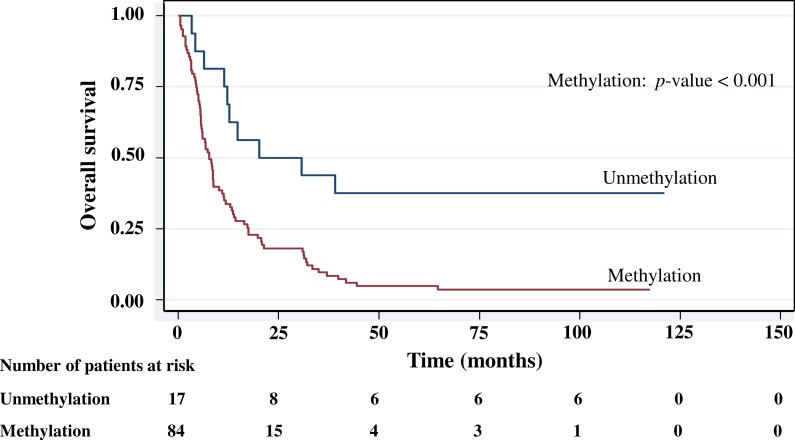
Survival curve of gastric cancer by *RASSF1A* methylation status (*n* = 101).

**Figure 6:**
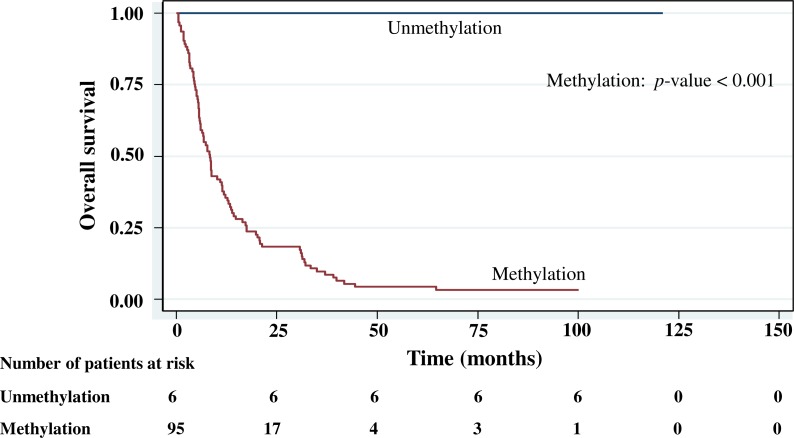
Survival curve of gastric cancer by *PCDH10* methylation status (*n* = 101).

**Figure 7:**
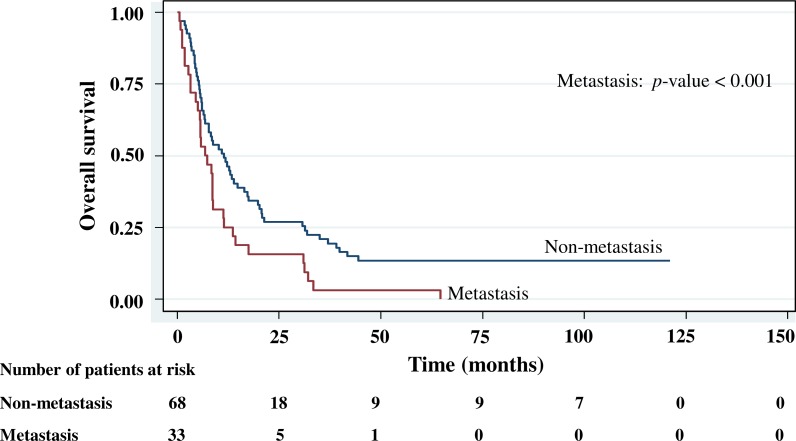
Survival curve of gastric cancer by metastasis status (*n* = 101).

The cumulative survival probabilities at 1, 3, 6, and 9 months were 96.0%, 86.9%, 63.6%, and 46.5%. respectively, and the 1, 2, 3, 4, and 5 year survival probabilities were 40.4%, 23.2%, 15.2% 10.1%, and 10.1%, respectively. The median survival time of stomach cancer after diagnosis was 8.07 months (95% CI [6.0–10.2]). Figures 4–7 shows the Kaplan–Meier curves for factors assessed for association with survival: stage, presence of metastasis, and *PCDH10* and *RASSF1A* methylation.

With histology staging by the TNM classification, stage IV had significantly reduced median survival time (8.6 months, *p* < 0.05) when compared with the lower stages (IA, IB, II, IIIA, IIIB). Similarly, with patients who had a metastasis, a low median survival time was noted (7.3 months, *p* < 0.01). With *RASSF1A* methylation, there was a large reduction of median survival time (7.8 months as compared with 20.2 months in the unmethylation group, *p* < 0.001). The median survival time with positive *PCDH10* methylation was just 8.4 months. With the *PCDH10* non-methylation patients no mortality was encountered.

Table 1 also shows the hazard ratios. In the multivariate analysis based on stepwise Cox proportional hazards regression, only *RASSF1A* methylation and stage proved to be significant independent risk factors for survival. However, no analysis of the *PCDH10* methylation data was conducted because of the very low number in the unmethylation group (*n* = 6) compared with the methylation group (*n* = 95). The outcome for this gene therefore remains inconclusive. There were no associations between gastric cancer survival and gender, age, anatomical region of cancer, histopathology, histology grading, or metastasis.

### Association of *RASSF1A* and *PCDH10* methylation status in cell-free DNA with clinicopathological parameters of gastric cancer patients

Table 2 summarizes the association of *RASSF1A* and *PCDH10* methylation status with various clinicopathological parameters. The aberrant methylation status in both genes was significantly associated with histology grading (differentiation of tumor), TNM stage (stage IV) and lymph node metastasis (*p* < 0.05). Gender, age, region of cancer, and histopathology were not significantly associated (*p* > 0.05) with either the *RASSF1A* or the *PCDH10* promoter methylations.

**Table 2:** Clinical characteristics of 101 gastric cancer patients with and without *RASSF1A* and *PCDH10* methylation in plasma DNA.

Clinical variables	*RASSF1A*		*p*-value	*PCDH10*		*p*-value
		
	Methylated (*n* = 84)	Non-methylated (*n* = 17)		Methylated (*n* = 95)	Non-methylated (*n* = 6)	
Gender			0.99			0.69
Female	37 (44.05%)	7 (41.18%)		42 (44.21%)	2 (33.33%)	
Male	47 (55.95%)	10 (58.82%)		53 (55.79%)	4 (66.67%)	
Age (years)			1.00			0.69
<50	35 (41.67%)	7 (41.18%)		39 (68.42%)	3 (50.00%)	
>50	49 (58.33%)	10 (58.82%)		56 (31.58%)	3 (50.00%)	
Region of cancer			0.34			1.00
Upper site and Body	19 (22.62%)	6 (35.29%)		24 (25.26%)	1 (16.67%)	
Lower site	41 (48.81%)	7 (41.18%)		45 (47.37%)	3 (50.00%)	
Not specific	24 (28.57%)	4 (23.53%)		26 (27.37%)	2 (33.33%)	
Histopathology (Adenocarcinoma)			1.00			1.00
NOS	58 (69.05%)	12 (70.59%)		66 (69.47%)	4 (66.67%)	
Intestinal and diffuse type	26 (30.95%)	5 (29.41%)		29 (30.53%)	2 (33.33%)	
Histology grading (Goseki classification)			0.001^***^			0.017^**^
Not specific	14 (16.67%)	7 (41.18%)		17 (17.89%)	4 (66.67%)	
Group I–IV	70 (83.33%)	10 (58.82%)		78 (82.11%)	2 (33.33%)	
Stage grouping (TNM classification)			0.002^**^			0.023^**^
Not specific	13 (15.48%)	10 (64.71%)		20 (21.05%)	3 (50.00%)	
I–III	17 (20.24%)	6 (29.41%)		20 (22.11%)	3 (50.00%)	
IV	54 (64.29%)	1 (5.88%)		55 (56.84%)	0 (0%)	
Metastasis			0.005^***^			0.002^***^
No	27 (39.29%)	12 (11.76%)		33 (34.74%)	6 (100.0%)	
Yes	57 (60.71%)	5 (88.24%)		62 (65.26%)	0 (0%)	

**Notes.**

*** *p* < 0.001.

** 0.001 < *p* < 0.01.

## Discussion

Although there are many studies related to epigenetic alterations associated with gastric cancer, the accuracy of molecular mechanisms between gastric cancer carcinogenesis and progression remains unclear. A specific prognostic biomarker in tumor tissues is required to predict disease progression in clinicopathological terms (Fu et al., 2015).

Cell-free DNA from cancer cells, one of non-invasive biomarkers in gastric cancer diagnosis and prognosis, is released to serum (Esposito et al., 2014). Many studies have also observed that the methylation level of *PCDH10* and *RASSF1A* is closely related to gastric cancer tissues, but there are only a few reports of these methylation levels in cell-free DNA. In the present study, the rates for DNA methylation in controls were very low (below 6%). This was in clear contrast to the 94 and 83% found for gastric cancer cases for *PCDH10* and *RASSF1A*, respectively.

This finding is consistent with earlier studies using tumor tissue (Otani et al., 2013; Jin, Jiang & Wang, 2015; Tahara & Arisawa, 2015; Yu et al., 2009; Yu et al., 2010) and provides the first such demonstration in Thailand. Furthermore, the high levels of *PCDH10* and *RASSFIA* methylations in plasma samples from cases suggest an ideal new biomarker for gastric cancer. The methylation process is accessible, repeatable, and noninvasive. This study used MSP to evaluate methylation status based on bisulfite reactivity, and bisulfite treated DNA was confirmed. MSP provides a sensitive, quick, and cost-effective test for the methylation status of CpG dinucleotides in a CpG island, making the technique applicable for high throughput analysis of clinical samples (Herman et al., 1996; Shanmuganathan et al., 2013; Wani & Aldape, 2016).

Yu et al. (2010) reported that *PCDH10* methylation was detected in 82% (85 of 104) of gastric tumors whereas it was found in only 37% (38 of 104) of paired non-tumor tissues (*p* < 0.001); while the study of Li et al. (2012a); Li et al. (2012b) showed an even a higher rate of hypermethylation at 86% of gastric cancer tissues and gastric cancer cell lines of *PCDH10*. Regarding *RASSF1A*, the reports provided by Joo et al. (2015) stated that 26% to 66.1% of *RASSF1A* methylation rates occurred in gastric cancer cell lines or tissues (Shi et al., 2014; Qu, Dang & Hou, 2013; Ye et al., 2007). Shi et al. (2014) assessed the association of *RASSF1A* promoter methylation with gastric cancer risk in a comprehensive meta-analysis and documented a significant linkage (*OR* = 12.67; 95% CI [8.12–19.78]; *p* < 0.001).

However, with *PCDH10*, none of the studies about cell-free DNA methylation levels have been measured in blood samples, but in *RASSFIA* methylation was found in 50 (68.5%) of the serum samples of 73 gastric cancers (Balgkouranidou et al., 2015) and, in another study,16 (34%) of 47 gastric cancers (Wang et al., 2008). Consistent with Kwon et al. (2012), disease staging is an important factor affecting the survival of gastric cancer patients, especially in terms of the advanced stages of the disease (Choi et al., 2015; Kwon et al., 2012; Jung et al., 2013). Our finding that the median survival time of more than 50% of the recruited patients at the severe stage of the disease was about 8 months after diagnosis is consistent with this. Likewise, the patients with metastasis, *RASSF1A* and *PCDH10* methylations also died during the first year. Notably in our finding, the methylation of *RASSF1A* and *PCDH10* was found higher in late-stage patients and were correlated with metastasis and histology.

Our research is the first report about an investigation into DNA methylation and clinicopathological characteristics associated with the survival of gastric cancer patients in a Thai population. Our analyses showed that aberrant *PCDH10* and *RASSF1A* promoter methylation in plasma DNA was associated with shorter overall survival. The occurrence of *PCDH10* and *RASSF1A* methylations in plasma DNA provides additional information with clinical relevance. For our study we recruited patients mainly from rural areas who attended two large regional hospitals. For most of them we found that a presence of gastric cancer was at the severe stage. Methylation detection is useful in drawing attention to extent of development of the cancer such as tumor differentiation, stage, and distant metastasis. This suggests that it may be useful in the clinical application of screening and diagnosis of gastric cancer.

Regarding *RASSF1A* methylation and survival of gastric cancer patients, different studies have reported a link with poor prognosis (Dammann et al., 2003; Grawenda & O’Neill, 2015). As expected, we found that tumor characteristics, such as stage of cancer and metastasis, were inversely linked to survival, although in the case of metastasis this was confirmed in our multivariate analysis. In line with other studies, stage of disease emerged as an important factor affecting survival of gastric cancer patients (Yamashita et al., 2007; Kwon et al., 2012; Jung et al., 2013; Choi et al., 2015; Kim et al., 2015; Huang et al., 2015).

## Conclusions

Gastric cancer is associated with methylated *PCDH10* and *RASSF1A*. In addition, only *RASSF1A* methylation and stage IV were found to be major factors having a direct effect on the survival of Thai gastric cancer patients. Whether the two genes can potentially be used as candidate clinical biomarkers still requires further validation in large scale prospective studies.

##  Supplemental Information

10.7717/peerj.2112/supp-1 Data S1 Data analysis Data of participants.

## Additional Information and Declarations

### Data Availability

The following information was supplied regarding data availability: The raw data has been supplied as a Data S1.
